# *Candida intermedia* Supplementation Enhances Immune Response and Modulates the Gut Microbiome in SARS-CoV-2 Vaccinated Mice

**DOI:** 10.3390/jof11090685

**Published:** 2025-09-20

**Authors:** Renan E. A. Piraine, Neida L. Conrad, Vitória S. Gonçalves, Jeferson V. Ramos, Júlia L. Froldi, Fausto Almeida, Fábio P. L. Leite

**Affiliations:** 1 Microbiology Laboratory, Technological Development Center, Federal University of Pelotas, Pelotas 96010-610, RS, Brazil; conradneida@gmail.com (N.L.C.); vitoriasgon@gmail.com (V.S.G.); jeff.bioinfo@gmail.com (J.V.R.); fleivasleite@gmail.com (F.P.L.L.); 2 Department of Biochemistry and Immunology, Ribeirão Preto Medical School, University of São Paulo, Ribeirão Preto 14040-900, SP, Brazil; julia_froldi@hotmail.com (J.L.F.); fbralmeida@usp.br (F.A.)

**Keywords:** probiotics, non-*Saccharomyces*, immune system, vaccination, yeasts

## Abstract

Non-*Saccharomyces* yeasts are emerging as promising new probiotics with a beneficial effect equal to or greater than the reference probiotic yeast, *Saccharomyces boulardii*. *Candida intermedia*, a non-*albicans* species not considered a common human pathogen, previously demonstrated probiotic potential. In this work, our objective was to evaluate the immunomodulatory effects of *C. intermedia* ORQ001 in mice vaccinated with inactivated SARS-CoV-2, seeking further evidence of its probiotic activity. Murine macrophages were stimulated with *C. intermedia*, followed by mRNA transcription analysis via qPCR. Mice were supplemented with *C. intermedia* prior to SARS-CoV-2 vaccination. Antibody production was assessed by ELISA, and fecal microbiomes were analyzed using next-generation sequencing. *C. intermedia* significantly increased *Il4* and *Il13* expression while decreasing *Stat3* in macrophages. Splenocytes from supplemented mice exhibited elevated transcription levels of *Tnf*, *Ifng*, *Il4*, *Bcl6*, and *Stat3* after exposure to stimulatory molecules. These mice showed increased levels of anti-SARS-CoV-2 IgG and sIgA isotypes, along with higher abundances of *Bacteroides* spp. and *Clostridium* spp. in their gut microbiome. In conclusion, *C. intermedia* supplementation modulated the expression of key immune-related genes and enhanced humoral responses in mice. Furthermore, its influence on gastrointestinal microbiota suggests a synergistic effect on vaccine immunogenicity. These findings support the potential of *C. intermedia* as a novel probiotic candidate with immunomodulatory properties applicable to vaccine adjuvanticity.

## 1. Introduction

Probiotics are live microorganisms, bacteria or fungi that are administered to a host in order to promote a positive effect on health, primarily by modulating immune system-related functions [1]. *Saccharomyces boulardii* has been commercialized and studied since the 1950s and is currently the best-characterized yeast in terms of its probiotic activity, safety, and application in the treatment of chronic gastrointestinal diseases and conditions [2]. Probiotic microorganisms positively impact intestinal surface integrity, producing antimicrobial compounds and metabolites that hinder pathogens’ growth and compete for binding sites in the intestinal mucosa [1].

Non-*Saccharomyces* yeasts emerge as possible new probiotics with a beneficial effect equal to or greater than that observed for *Saccharomyces* yeasts [3]. *Candida intermedia* was already described 30 years ago in fermented dairy foods [4], and recently had its genome sequenced [5,6]. Some strains of *C. intermedia* have demonstrated the ability to decrease or suppress the growth of foodborne pathogens such as *Listeria monocytogenes* [3,7], in addition to being responsible for the production of antimicrobial peptides that affect other yeasts [8]. In a previous study [9], we identified in vitro some potential probiotic characteristics in the isolate *C. intermedia* ORQ001, such as high levels of auto-aggregation, co-aggregation with pathogenic Gram− and Gram+ bacteria, and a low decrease in cell viability after exposure to gastrointestinal tract (GIT) conditions. Based on these results, we focused on the development of new studies regarding the probiotic potential of this yeast. Also, our group has reported an immunomodulatory effect for *S. cerevisiae* and *S. boulardii* improving immune responses to vaccines [10,11].

The administration of vaccines against SARS-CoV-2 became a global priority during the COVID-19 pandemic, leading to the development of over 100 vaccines employing various strategies and technologies. Inactivated vaccines have been adopted for mass vaccination and phase 1 and 2 clinical trials have consistently demonstrated a low rate of adverse reactions and notable immunogenicity with potent protection against the virus challenge in non-human primates [12]. However, studies have demonstrated that estimated vaccine efficacy of inactivated SARS-CoV-2 vaccines is still variable, mainly for controlling SARS-CoV-2 infection and symptomatic COVID-19 [13,14]. One way to improve vaccine efficacy is to supplement the host with probiotics [10,15,16,17]. Probiotics have been implied to be capable of immunomodulation by enhancing antibody production, increased phagocytic activity, and changes in cytokine expression [18,19,20].

Thus, the present study aims to evaluate the *C. intermedia* ORQ001 probiotic potential in the immune vaccinal response, using an experimental vaccine composed by inactivated SARS-CoV-2 in a murine model. To the best of our knowledge, this is one of the first reports exploring a non-conventional yeast as an immunobiotic candidate in the context of coronavirus vaccination. By combining analyses of immune activation and gut microbiome modulation, this work provides original insights that may broaden the understanding of host–microbe interactions in vaccination and contribute to the development of innovative probiotic-based strategies to improve vaccine efficacy.

## 2. Material and Methods

### 2.1. Strains and Culture Conditions

*Candida intermedia* ORQ001 and *Saccharomyces boulardii* CNCM I-745 (Floratil ^®^, Rio de Janeiro, Brazil, a reference probiotic strain) were obtained from the microorganism bank of the Microbiology Laboratory in the Federal University of Pelotas (Rio Grande do Sul, Brazil). Yeasts were grown overnight in YM (Yeast and Malt Extract) medium (0.3% yeast extract, 0.3% malt extract, 0.5% peptone, and 1% glucose) at 30 °C under constant agitation of 150 rpm. Successive steps of propagation under the previous conditions were conducted to scale up the yeast cultures, to a final concentration of 1 × 10^8^ CFU/mL (Colony-Forming Units). Cells were concentrated by centrifugation at 2000× *g* for 10 min using a DAIKI DTR-16000 centrifuge (Ionlab, Araucária, Brazil), counted by sequential serial dilutions, and stored at 4 °C until their use.

### 2.2. RAW 264.7 Cells Culture and Stimulation with Yeasts

To evaluate the capacity of *C. intermedia* to stimulate cytokine transcription, we used the macrophage cell line RAW 264.7 (ATCC^®^ TIB-71^TM^). *S. boulardii* was also used in this experiment, as reference for a response mediated by a probiotic yeast. RAW cells were grown as monolayers according to Santos et al. [21]. Briefly, cells were incubated in Dulbecco’s Modified Eagle Medium (DMEM) supplemented with 10% (*v*/*v*) Fetal Bovine Serum (FBS) at 37 °C in a 90% humidity atmosphere with 5% CO_2_ until approximately 80% confluence in the culture plate.

Stimulation was performed at a yeast-to-RAW cell ratio of 10:1, following an adapted version of the protocol developed by Smith et al. [22]. For the assay, 1 × 10^7^ CFU/mL of live (yeast) cells per well were used as stimuli. RAW cells were exposed to stimulation for 20 h in DMEM supplemented with 10% (*v*/*v*) FBS, incubated at 37 °C in a humidified atmosphere containing 5% CO_2_. The same procedure was applied using heat-killed cells, cell-free supernatants (obtained by centrifugation of yeast cultures), and total yeast DNA (extracted from pelleted cells). As a negative control, RAW cells were maintained with DMEM only.

After the stimuli, the supernatant was discarded, and cells were collected with the TRIzol^®^ reagent (Sigma-Aldrich^®^, St. Louis, MO, USA) and stored at −70 °C. RNA extraction from RAW macrophages was performed according to the protocol provided by the manufacturer using the TRIzol method.

### 2.3. Mice Experimental Design

Ninety Balb/c mice of an age of 4–6 weeks were provided by the Central Animal Facility of the Federal University of Pelotas. Mice were divided into nine experimental groups with ten animals each, as shown in Table 1. Animal supplementation was performed once a day by oral administration (gavage) of 500 µL of yeasts *C. intermedia* or *S. boulardii*, at a concentration of 1 × 10^8^ CFU/mL. The supplementation was performed five days before each vaccine dose, based on a protocol for short-term supplementation suggested by Santos et al. [23]. For the non-supplemented group, the same volume (500 µL) of sterile PBS was administered. Throughout the experiment, the animals were fed ad libitum with a commercial diet free of chemotherapeutics (Nuvilab^®^ CR1 irradiated, Seoul, Republic of Korea). All procedures performed followed the guidelines of the Brazilian College of Animal Experimentation (COBEA) and were approved by the Ethics Committee on the Use of Animals at UFPel (CEEA n° 011015/2022-75).

The experimental vaccine was elaborated with formaldehyde-inactivated SARS-CoV-2 virus (kindly provided by Prof. Fernando Spilki, from Feevale University), at a concentration of 1 × 10^6^ PFU (Plate-forming Units), added with 10% aluminum hydroxide (Sigma, St. Louis, MI, USA) as an adjuvant. The mice were inoculated subcutaneously (100 µL) twice, with an interval of 21 days between vaccinations. For control group mice (unvaccinated groups), they were inoculated with a suspension composed of 100 µL of PBS with 10% aluminum hydroxide.

Blood samples were collected by the submandibular puncture on days 0, 7, 14, 21, 28, 35, and 42, with serum being separated by centrifugation at 5000× *g* for 5 min and then stored at −20 °C until analysis. Mice from groups A, B, and C were euthanized on day 0, while animals from the other groups were sacrificed on the 42nd day (end of the experiment). A graphical scheme of the schedule containing all the procedures and time points is presented in Supplementary Material—Figure S1.

#### 2.3.1. Total Specific Antibodies Anti-SARS-CoV-2

An indirect enzyme immunoassay (ELISA) was used to detect anti-SARS-CoV-2 antibodies in the sera of vaccinated mice. Briefly, microtitre plates (96-well, Cral^®^, São Paulo, Brazil) were coated with inactivated SARS-CoV-2 virus (1 × 10^5^ PFU/mL) diluted in 0.1 M Carbonate-Bicarbonate buffer pH 9.8 overnight at 4 °C, washed three times with PBS-T (Phosphate-buffered saline +0.05% Tween 20), and then incubated for 2 h at 37 °C with 100 µL/well of powdered milk 5% diluted in PBS. After a new step of PBS-T washing, pooled sera from each group were added in triplicate at a 1:100 dilution, for 2 h at 37 °C. Anti-mouse IgG secondary antibodies conjugated with Horseradish Peroxidase (HRP) (Sigma-Aldrich^®^) at a dilution of 1:5000 were applied after five PBS-T washes and incubated again at the same conditions as previously described. The ELISA plates were washed again, and a substrate buffer (0.4 mg ortho-phenylenediamine, 15 µL H_2_O_2_, and 0.1 M phosphate citrate buffer pH 4.0) was added with 100 µL/well to reveal the reaction. After 15 min in the dark at room temperature (~25 °C), 50 µL/well of 2 N sulphuric acid to stop the reaction. Absorbance was measured in a microplate reader (Thermoplate^®^, Troy, MI, USA) with a 492 nm filter. Moreover, for antibody titration, using the same serum samples (D28, D35, and D42), two-fold dilutions were made in a range of 1:100 to 1:12,800. The cut-off for antibodies titers was defined as the absorbance of day 0 plus the standard deviation.

For the detection of sIgA (secretory IgA isotype) in fecal samples, we conducted the ELISA test protocol described by Santos et al. [21]. Initially, fecal samples from all 10 animals per group were pooled to obtain a single composite sample per group (*n* = 1 pool/group), ensuring sufficient volume for downstream sequencing. Subsequently, 0.1 g of the pooled fecal samples collected on experimental day 42 were resuspended in 1 mL of 1% PBS containing 1 mM phenylmethylsulfonyl fluoride (PMSF, Sigma-Aldrich^®^) and 1% bovine serum albumin (BSA), followed by thorough homogenization by vortexing. Thus, these samples were diluted with PBS-T + 5% powdered milk in a 1:2 ratio, then added (100 µL/well) over a plate coated with the SARS-CoV-2 virus. Specific sIgA antibodies to the SARS-CoV-2 virus were detected with goat anti-mouse IgA alpha-chain + HRP (Abcam^®^, Cambridge, UK), diluted to 1:1000. Incubation periods, washing steps, and solutions used for ELISA tests described before were also used for sIgA ELISA. For all ELISA assays (both IgG and sIgA isotypes), each pooled serum sample was tested in triplicate wells and evaluated on at least two independent plates to ensure consistency and accuracy in the detection of specific antibody production.

#### 2.3.2. Splenocytes Cytokines Transcription

To evaluate the effect of yeast supplementation on the cellular response of the supplemented mice, the quantitative PCR method (qPCR) was used to amplify fragments of cytokine genes, transcription factors, and receptors in the cDNA obtained from mRNA. At day 0, just after the first five days of supplementation, spleens from mice in groups A, B, and C were pooled in duplicates of five each, to evaluate the immediate effect on the cytokine transcription after the period of administration of *C. intermedia* and *S. boulardii*. The spleens were removed from animals and processed, and then their cells (splenocytes) were suspended in a balanced HANK’S solution (without Ca^2+^ and Mg^2+^ ions). Cells were centrifuged and pellet suspended in lysis solutions (0.8% ammonium chloride), followed by a new step of wash and suspension in RPMI 1640 (Sigma-Aldrich^®^) with 10% FBS (Cultilab^®^, Campinas, Brazil), totaling a standard concentration of 2 × 10^6^ cells/mL. These cells were cultivated in 24-well plates (Kasvi^®^, São José dos Pinhais, Brazil), 1 mL per well, and incubated for 24 h at 37 °C with 5% CO_2_. After this period, the medium was renewed, and cells were stimulated in different ways: Zymosan (100 µg), and Lipopolysaccharide (LPS) (10 µg). The stimuli were carried out during 18 h of incubation for 24 h at 37 °C with 5% CO_2_, then the supernatant was discarded, cells were collected, and the RNA extraction from splenocytes was performed using the TRIzol^®^ method.

At the end of the experiment (42nd day), duplicates of five spleens from mice from each of the other groups (D, E, F, G, H, and I) of vaccinated and unvaccinated animals, supplemented or not, were used to obtain splenocytes and evaluate cytokine transcription patterns when stimulated with 1 × 10^5^ PFU/mL of inactivated SARS-CoV-2 virus. Incubation and stimulus conditions (time, temperature, culture medium), were maintained as previously described, and RNA extraction was performed as done before using TRIzol^®^.

#### 2.3.3. Quantitative Real-Time PCR Analysis of Cytokines and Transcription Factors Genes

The reaction for cDNA synthesis was performed using 400 ng of RNA, following the instructions available in the High-Capacity cDNA Reverse Transcription Kit (Applied Biosystems^®^, Foster City, CA, USA). Quantitative PCR was conducted in a Stratagene Mx3005P real-time PCR system (Agilent Technologies^®^, Santa Clara, CA, USA) to analyze the relative transcription of the cytokine genes *Il2*, *Il4*, *Il12*, *Il13*, *Il23*, *Tnf*, *Ifng*, and transcription factors *Nfκb*, *Bcl6*, and *Stat3*. The *β*-actin gene (*Actb*) was used as endogenous reference control. Primer sequences were listed on the Supplementary Material—Table S1. Conditions used in the qPCR reactions have been described previously [24]. All samples were analyzed in triplicate. Relative expressions were calculated by comparing Threshold Cycle (Ct) values of *Actb* and targeted genes with the non-supplemented group, according to the 2^−ΔΔCT^ method described by Livak and Scmittgen [25].

#### 2.3.4. Gastrointestinal Microbiome Evaluation

Fecal samples collected on day 0 (after 5 days of yeast supplementation) were kept cold and sent to Neoprospecta^®^ (Florianópolis, Brazil) for processing. DNA extraction, purification, and library preparation were performed by Neoprospecta according to the methods described by Christoff et al. [26]. Next-generation sequencing (NGS) was then conducted to characterize the gastrointestinal microbiome. The amplicons were sequenced in paired-end mode (2 × 300 bp) with Miseq Reagent Kit V3 R (600 cycles) on the Miseq Sequencing System platform (Ilumina^®^, San Diego, CA, USA). Sequencing of the V3/V4 regions of the ribosomal RNA gene was performed with primers 341F (sequence CCTACGGGRSGCAGCAG), and 806R (sequence GGACTACHVGGGTWTCTAAT). Negative and positive controls were used during all processes. The raw data obtained from sequencing were submitted to quality control, taxonomic classification, visualization, and description of communities using the pipeline Sentinel (Neoprospecta^®^) and Knomics-Biota system [27]. Taxonomic classification was evaluated based on the SILVA reference database (v138) [28]. Using the *R* programming language and *microbiome* package, the OTUs (Operational Taxonomic Units) table was normalized by “clr-transformation” to calculate alpha and beta diversities [29]. Alpha diversity was determined using Observed, Shannon, Simpson, InvSimpson, Fisher, and Evenness indices. The Kruskal–Wallis test was inferred to verify significant differences among groups (*p* < 0.05). Beta diversity was estimated by Principal Component Analysis (PCA), using the Aitchison matrix [30].

### 2.4. Statistical Analysis

Serology data and those related to cellular response were analyzed by analysis of variance (2 way ANOVA) with Dunnett’s multiple comparison test, and the statistical difference was determined if the *p* < 0.05. All analyzes were mainly performed in the statistical software GraphPad Prism 11 v.7.

## 3. Results

### 3.1. Immunostimulatory Activity of Viable Yeast Cells and Their Derivatives on RAW 264.7 Cells

**Figure 1:**
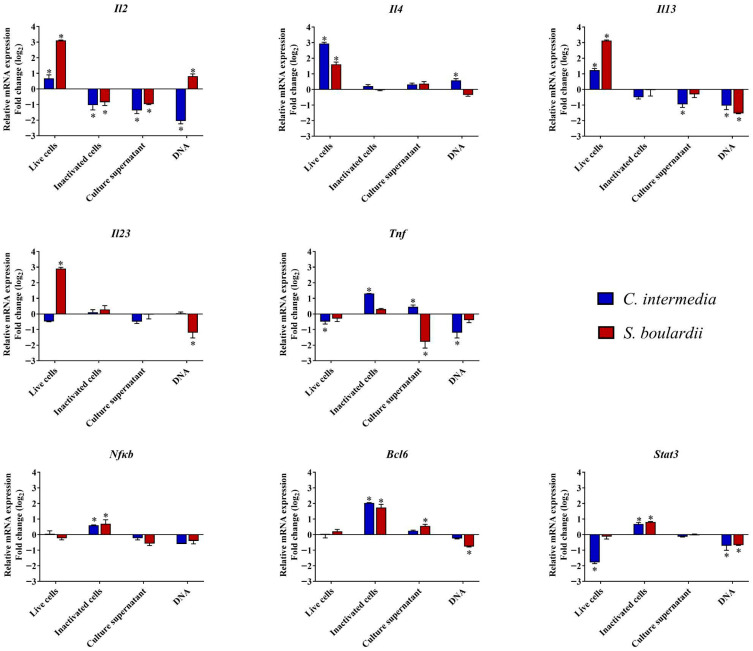
Gene transcription of cytokines *(Il2*, *Il4*, *Il13*, *Il23*, *Tnf*) and transcription factors *(Nfκb*, *Bcl6*, *Stat3*) in RAW 264.7 macrophages stimulated with yeasts and their derivatives. The data represent mRNA transcription levels in macrophages co-incubated with viable and non-viable yeast cells of *C. intermedia* ORQ001 and *S. boulardii* CNCM I-745, as well as with the respective culture supernatants and fungal DNA. Relative mRNA expression was normalized to β-actin (*Actb*) transcript levels. Data are presented as mean ± standard deviation (SD). The statistical analysis was performed with 2-way ANOVA. The symbol “*” indicates a significant difference (*p* < 0.05) compared to unstimulated RAW 264.7 cells (basal expression). Expression levels were tested in duplicates and evaluated on at least two independent plates.

Live cells of *C. intermedia* were capable of a significant stimulation (*p* < 0.05) of the *Il4* and *Il13* mRNA transcription in RAW cells. Moreover, we observed that *Stat3* transcription was downregulated. Meanwhile, viable cells of *S. boulardii* stimulating RAW cells were able to upregulate the mRNA transcription of cytokines *Il2*, *Il4*, *Il13*, and *Il23* (Figure 1). While the primary focus was on the immunomodulatory effects of live yeast cells, we also evaluated the responses of macrophages to fungal cell derivatives. Treatment with heat-killed cells, culture supernatant, and fungal DNA revealed differential effects, suggesting that certain immunomodulatory responses are dependent on the presence of metabolically active live cells.

Inactivated cells of *C. intermedia* were not able to stimulate an increase in *Il4* and *Il13* transcription, but an upregulation was identified for *Tnf*. Also, a potentiation in the mRNA transcription of *Bcl6* was observed. *S. boulardii* killed cells caused a downregulation of *Il2* and an upregulation of *Bcl6*, which contrasts with that observed when live cells of this yeast were used as a stimulus for RAW 264.7 macrophages. We observed that *C. intermedia* culture supernatant induced a decrease in *Il2* and *Il13* levels, as well as the supernatant of *S. boulardii* culture, which resulted in a decrease in *Il2* and *Tnf* transcription. Finally, we found that DNA extracted from *C. intermedia* stimulated a significant inhibition of *Il2* mRNA transcription, with less intensity for *Il13* and *Stat3*. Likewise, *S. boulardii* DNA has also been shown to impact the mRNA transcription of some genes, positively stimulating *Il2* and diminishing *Il13*, *Il23*, *Bcl6* and *Stat3*.

### 3.2. Modulation of Cytokine Gene Expression in Splenocytes Stimulated with Yeasts

**Figure 2:**
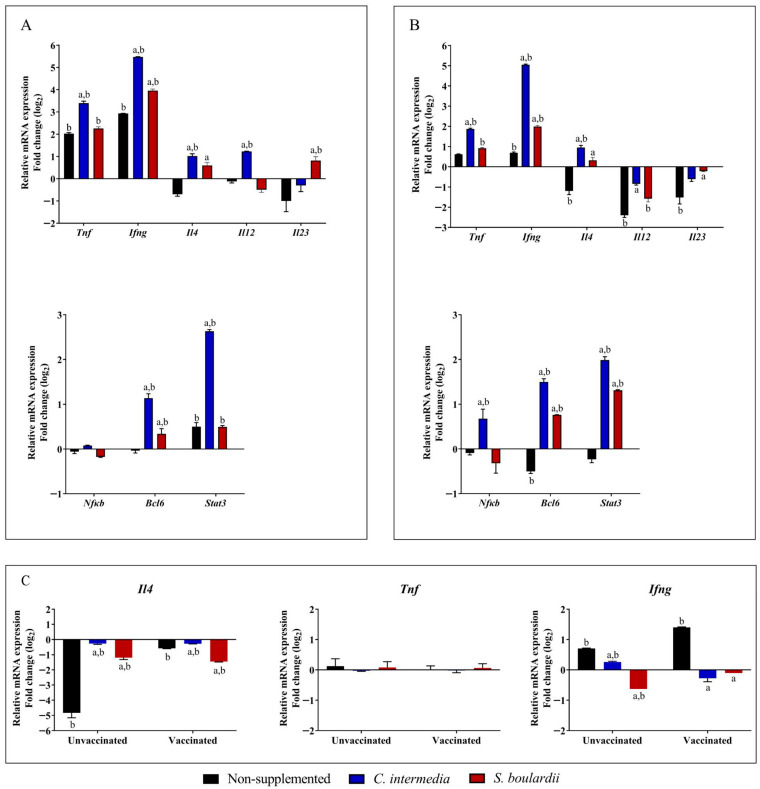
Gene transcription of cytokines and transcription factors during splenocyte stimulation. Splenocytes were obtained from animals supplemented for five days with *C. intermedia* or *S. boulardii*, or from non-supplemented controls (*n* = 5/group). Cells were cultured in vitro and stimulated with Zymosan (**A**) or LPS (**B**). Relative mRNA expression of *Tnf*, *Ifng*, *Il4*, *Il12*, *Il23*, *Nfκb*, *Bcl6*, and *Stat3* was normalized to *Actb* expression. Additionally, after the 42-day experimental schedule, splenocytes from supplemented or non-supplemented animals (*n* = 5/group), whether vaccinated or not, were subjected to in vitro stimulation with SARS-CoV-2 (**C**). mRNA expression of pro-inflammatory (*Ifng*, *Tnf*) and anti-inflammatory (*Il4*) cytokines was evaluated. Data are presented as mean ± standard deviation (SD). The statistical analysis was performed with 2-way ANOVA. The letter “a” indicates a significant difference (*p* < 0.05) compared to the non-supplemented group, while “b” indicates a significant difference (*p* < 0.05) compared to unstimulated splenocytes (basal expression levels). Expression levels were tested in duplicates and evaluated on at least two independent plates.

After five days of supplementation with *C. intermedia* and *S. boulardii*, splenocytes from animals from each group were collected and cultured in vitro. These cells were stimulated with Zymosan and LPS, and an increased mRNA transcription for *Tnf*, *Ifng*, *Il4*, and transcription factors *Bcl6*, *Stat3* was observed in splenocytes from mice supplemented with both yeasts, especially for the non-*Saccharomyces* yeast (Figure 2). Noteworthy that even showing a similar pattern in cytokines upregulation, splenocytes from mice supplemented with *C. intermedia* showed higher transcription levels when stimulated with Zymosan (Figure 2A) and LPS (Figure 2B). The most upregulated gene was *Ifng*, for which an increase of 20.4-fold was detected in LPS-stimulated cells and 5.8-fold after stimulation with Zymosan.

Splenocytes collected on day 42 from vaccinated and unvaccinated mice, whether supplemented or not, were stimulated with inactivated SARS-CoV-2, and the mRNA expression of *Ifng*, *Tnf*, and *Il4* is presented in Figure 2C. Splenocytes from unvaccinated and non-supplemented animals stimulated with SARS-CoV-2 exhibited a markedly decreased *Il4* mRNA expression, accompanied by an upregulation of *Ifng*. Upon vaccination, this cytokine expression profile was maintained; however, *Il4* downregulation was attenuated, and *Ifng* expression levels were further enhanced. In animals supplemented with *C. intermedia*, both vaccinated and unvaccinated groups displayed basal mRNA expression levels of *Il4* and *Ifng*. A similar expression pattern was observed in the group supplemented with *S. boulardii*; however, a significant downregulation of *Ifng* was detected in unvaccinated animals. In all groups, *Tnf* mRNA expression by splenocytes was maintained at baseline levels. The fold-change values of mRNA expression for cytokines and transcription factors, from both macrophage and splenocyte assays are presented in the supplementary material, including a representation of upregulation and downregulation of the evaluated genes (Supplementary Material—Tables S2–S7).

### 3.3. Dynamics of IgG Production and sIgA Detection

**Figure 3:**
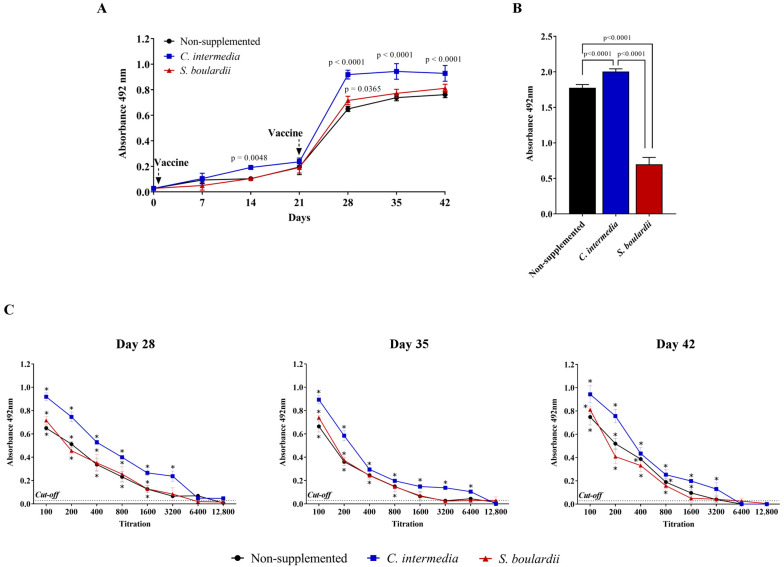
Humoral immune response of the animals non-supplemented (control) and supplemented with *C. intermedia* and *S. boulardii* and vaccinated with inactivated SARS-CoV-2. (**A**) Dynamic of serum IgG production, analyzed each seven days from pooled sera of the experimental groups. (**B**) sIgA detection on pooled fecal samples of mice in the 42nd experimental day. (**C**) Titration of total IgG levels on samples from the 28th, 35th, and 42nd day post immunization. The data represent the means (±standard deviation) of the absorbance values (492 nm) obtained by indirect ELISA. Each pooled serum sample was tested in triplicate wells and evaluated on at least two independent plates. The statistical analysis was performed with 2-way ANOVA. When statistical differences were detected, the exact *p*-values were added to the graphs, or alternatively indicated with the symbol “*” when *p* < 0.05 (specifically in the antibody titration graphs) compared to the non-supplemented group (PBS).

All groups of mice responded to the vaccine producing specific IgG anti-SARS-CoV-2. The *C. intermedia*-supplemented group showed significantly higher antibody levels (*p* < 0.05) than the other two groups after 14 days of the first vaccine dose and until the end of experimentation period. After the vaccine boost at day 28 (seven days after the vaccine boost) a more significant of ~4-fold response for specific IgG anti-SARS-CoV-2 was observed in *C. intermedia*-supplemented mice, whereas the other groups responded with ~3.0 folds, and those levels were kept until the end of the experimentation (Figure 3A). Evaluating the presence of sIgA in the fecal samples from supplemented and control animals, we observed that the *C. intermedia* supplemented mice showed a significant (*p* < 0.05) higher level of sIgA than the other groups on the last experimental day. Worth noting was the *S. boulardii* supplemented groups showing the lowest sIgA levels even lower than the control non-supplemented mice (Figure 3B).

In the IgG titration assay (Figure 3C), sera collected on day 28 from the non-*Saccharomyces* group exhibited an antibody titer of 3200, which was twice as high as that observed in the non-treated (non-supplemented) group (1600). On day 35, the *C. intermedia*-supplemented group maintained a titer of 6400, representing an eightfold increase compared to the non-supplemented animals (800). On the final experimental day (day 42), only the *C. intermedia*-supplemented group sustained the titer of 3200, while the non-supplemented group showed 1600. Sera from animals supplemented with *S. boulardii* showed the same titers as the non-supplemented group on days 28 and 35; however, a reduction was observed on day 42, with the *S. boulardii* group presenting a titer of 800.

### 3.4. Gastrointestinal Tract Microbiomes of Supplemented and Non-Supplemented Animals

**Figure 4:**
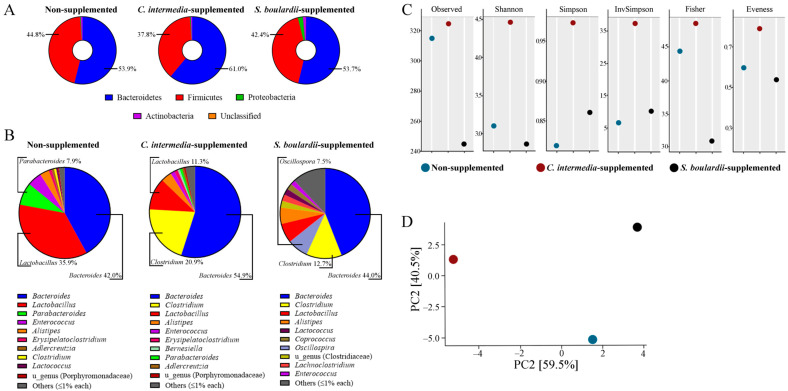
Microbiome analysis of the pooled fecal samples from non-supplemented, *C. intermedia*-supplemented, and *S. boulardii*-supplemented animals (*n* = 10/group). After a 5-day schedule of supplementation, fecal samples were collected from different animals in each experimental group, pooled, and submitted for microbiome analysis. (**A**) The most abundant bacterial genera in the sequenced samples, emphasizing the top 3 in each sample. (**B**) Bacterial abundance at the phylum level. (**C**) Alpha-diversity of the microbiomes, demonstrated by Observed, Shannon, Simpson, InvSimpson, Fisher, and Evenness indices, (**D**) Beta-diversity, estimated by Principal Component Analysis (PCA).

The microbiome of fecal samples from treated and non-treated groups revealed some important differences in bacterial composition, which were suggested to be related to supplementation with *C. intermedia* and *S. boulardii*. We observed in the control group (non-supplemented, with a normal diet) that the microbial environment on GTI is composed, at the phylum level, of Bacteroidetes (53.9%) and Firmicutes (44.8%), with less abundance (<1% each) for Actinobacteria and Proteobacteria. Analyzing the genera found, *Bacteroides* spp. (42.0%), *Lactobacillus* spp. (35.9%) and *Parabacteroides* spp. (7.9%) (Figure 4A) were the most abundant among the bacterial OTUs classified.

It was detected in the microbiome of the *C. intermedia*-supplemented group a similar profile for the 10 most abundant genera compared to the non-supplemented group; however, we observed a higher abundance for *Bacteroides* spp. (54.9%) and a shift in *Clostridium* spp. and *Lactobacillus* spp. OTUs abundance, which was 20.9% and 11.3%, respectively. Thus, this observation impacted the Bacteroidetes/Firmicutes balance, showing an abundance of 61% of Bacteroidetes and 37.8% of Firmicutes (Figure 4B). Meanwhile, the microbiome identified in the GTI sample obtained from *S. boulardii*-supplemented animals also showed a higher abundance of *Clostridium* spp. (12.7%), a similar level of *Bacteroides* spp. (44.0%), and a more pronounced presence of other genera with relevant abundance (≥1%) for the top 10 detected, such as *Oscillospira* spp. (7.5%). Although other genera were detected, the abundance of Bacteroidetes (53.7%) and Firmicutes (42.4%) was maintained in a similar proportion to that observed for the control group.

The alpha diversity of the microbiomes was measured by different indices. The species richness was detected through the observation of 244 species being identified in the non-supplemented group sample, while samples of *C. intermedia*-supplemented animals showed 315 and *S. boulardii*-supplemented animals showed 325 species detected (Figure 4C). While the non-supplemented group and the *C. intermedia*-supplemented group demonstrated in their sample’s close values for Shannon, Simpson, InvSimpson, and Evenness indices, the *S. boulardii*-supplemented group showed the highest values for these indices, as well as for Fisher (in which treated groups demonstrated closer values). In the beta-diversity analysis (Figure 4D), we observed that the microbiome of animals supplemented with *C. intermedia* was less altered when compared to non-supplemented animals, while this alteration was more significant for the group supplemented with *S. boulardii*.

## 4. Discussion

As part of the investigation into the probiotic adjuvanticity mechanism of *C. intermedia*, the murine macrophage cell line RAW264.7 was employed as an experimental model. The results obtained showed a significant increase in transcription for *Il4* and *Il13* when the cell was stimulated with live *C. intermedia*, with a transcription level for *Il4* of 2.5-fold higher than that observed for *S. boulardii*. *Il4* and *Il13* are the major effector cytokines produced by Th2 cells during type 2 immune responses, being critical for protective immunity against infections. These Th2 cytokines are able to induce macrophage proliferation and activation and facilitate antigen presentation through increased expression of MHC class II molecules [31]. *S. boulardii* was able to stimulate RAW264.7 to produce high transcription levels of *Il2* and *Il23*, differently than *C. intermedia*. IL-2 promotes the growth and development of peripheral immune cells, initiating a defensive immune response through the survival and division of regulatory T cells (Treg) and proliferation of cytotoxic T cells [32]. IL-23 has an immunoregulatory and pro-inflammatory role, sustaining cell-mediated responses focusing on intracellular infection elimination [32,33]. Our findings corroborate reports by Bazan et al. [34] that verified a response based on IL-12, IL-23, and IL-27 cytokines when stimulated with different yeast genera, including *Saccharomyces* spp. and *Candida* spp. Although extracellular vesicles (EVs) from *C. intermedia* have not yet been described, it is plausible that they contribute to host immune modulation. This hypothesis is supported by studies on other *Candida* species, whose EVs have been shown to carry immunologically active molecules capable of stimulating innate immune cells. Notably, *C. albicans* EVs can induce the production of anti-inflammatory and type 2-associated cytokines in macrophages and dendritic cells, suggesting a potential role in shaping a tolerogenic or Th2-skewed immune environment [35,36]. Thus, the potential role of *C. intermedia* EVs in immunomodulation warrants further investigation.

Probiotic yeasts, such as *S. boulardii* and potentially other non-conventional yeasts like *C. intermedia*, can modulate host immunity through the recognition of their conserved cell wall components by pattern recognition receptors (PRRs), including Toll-like receptors (TLRs) and C-type lectin receptors such as Dectin-1 [37]. The yeast cell wall is rich in β-glucans, mannans, and chitin, molecules that serve as microbe-associated molecular patterns (MAMPs) [38]. β-glucans are particularly recognized by Dectin-1, which is expressed on antigen-presenting cells such as dendritic cells and macrophages, and this interaction activates spleen tyrosine kinase (Syk) signaling pathways, leading to the production of cytokines such as IL-10, IL-6, IL-12, and TNF-α [37,39]. Simultaneously, mannoproteins can activate TLR2 and TLR4, contributing to the maturation of dendritic cells and the polarization of adaptive immune responses [40]. This dual engagement of PRRs not only promotes the differentiation of naïve T cells into Th1, Th17, or regulatory T cell subsets, depending on the cytokine context, but also enhances antigen presentation and the expression of costimulatory molecules [41]. Variations in cell wall composition of yeast species lead to differences in phagocytosis and levels of cytokines being produced by immune cell lines stimulated in vitro [42]. There are extensive variations in yeast cell walls when comparing different fungal species and strains, such as α-glucans in addition to β-glucans, and differences in concentration of chitosan, galactomannans, and melanin [43]. Although cell wall composition between *C. intermedia* and *S. boulardii* is comparable, Lozančić et al. [44] demonstrated significant differences in their genera regarding patterns of GPI-anchored and non-covalently attached proteins, cell wall thickness, permeability, and amounts of mannan and glucans.

The stimulation of macrophages by viable and non-viable cells depends on the cell structure of each yeast, cellular portions, yeast surface, internal cellular components, and actively secreted molecules (by live cells) to the extracellular environment [45]. In addition, the medium supernatant (cell-free), obtained after yeast culture, also may have metabolic byproducts that interact with immune system cells [46]. Although Smith et al. [45] did not find differences in cytokine-inducing properties among live, UV-irradiated, and heat-killed cells, in our study, a considerable variation (*p* < 0.05) in relative mRNA transcription was detected between stimuli with viable and non-viable cells. After a heat treatment associated with high pressure, cell inactivation occurs through membrane damage, loss of nutrients and ions, protein denaturation, and essential enzyme inactivation, which can lead to modifications in cell coarseness and roughness [47,48]. Cell inactivation demonstrated that the metabolic activity of viable cells influences the macrophage–yeast interaction, as evidenced by the reduced induction of mRNA transcription of cytokines such as *Il2*, *Il4* and *Il13*. Under these conditions, the previously observed upregulation was no longer detected; instead, a downregulation of *Il2* or maintenance of basal transcription levels for *Il4* and *Il13* was observed. While viable *C. intermedia* cells led to near-basal levels of *Tnf* and *Bcl6* mRNA transcription, inactivated cells stimulated a significant increase in their expression.

Modulation of vaccine response by *C. intermedia* supplementation was observed in the present study using an experimental vaccine composed of inactivated SARS-CoV-2. *C. intermedia* supplementation was able to improve the vaccine immunogenicity by promoting elevated levels of specific IgG antibody against SARS-CoV-2 as well as sIgA better than the control (non-supplemented mice) and *S. boulardii* group. The vaccine immune response in mice supplemented with *C. intermedia* was already significantly higher by day 14, compared with other groups. Of note was the prompt modulation on IgG levels in mice supplemented with *C. intermedia* after the first vaccine dose. It is possible to hypothesize that a single vaccine priming during a SARS-CoV-2 outbreak in individuals supplemented with *C. intermedia* might contribute to an immune response, potentially reducing disease severity, although this requires further validation. Supporting this concept, enhanced immune responses to SARS-CoV-2 following vaccination have previously been observed in mice orally administered the probiotic bacterium *Lactobacillus plantarum* GUANKE [49]. Moreover, probiotics have also been shown to positively influence seroconversion and seroprotection in humans vaccinated against other respiratory viruses, particularly influenza vaccines [50]. It is believed that immunomodulation mediated by the probiotic occurs during the primary vaccine antigen sensibilization, and the probiotic immune modulation generates an effective vaccine anamnestic response [16]. After the boost the *C. intermedia*-supplemented animals had a ~5-fold increase in the IgG levels, while the *S. boulardii* and control group had ~3.5- and 3.0-fold IgG levels increase, respectively. Additionally, IgG levels were kept the same for all groups until the end of the experiment.

The sIgA modulation in mice supplemented with *C. intermedia* was a very promising finding, since its level was significantly superior to the other groups, but principally higher than the levels observed in the *S. boulardii* group. These findings may be relevant since secretory IgA is predominant in mucosal surfaces and plays an important role in viral immunity; its potential contribution to enhanced SARS-CoV-2 neutralization has been suggested but is not yet fully established [51]. Klingler et al. [52] also described the importance of IgA antibodies; moreover, it was found that IgG1 and IgM have a strong contribution to SARS-CoV-2 neutralizing activity. Overall, the data suggest immune modulation but cannot be taken as proof of enhanced vaccine efficacy.

Cytokine secretion modulated by probiotic supplementation builds a suitable environment for interaction of the vaccine antigen and the immune system guiding the vaccine immune response [16,17,20]. In the present study, a similar mRNA transcription profile was observed in splenocytes from mice supplemented with *C. intermedia* and *S. boulardii* when stimulated with Zymosan and LPS, which are well-known immune response stimulants with different origins [53,54]. Comparing immunological responses after stimulation with these molecules permits the prediction of and helps identify the influence of yeast administration on animals’ immune systems in different scenarios. Splenocytes from *C. intermedia*-supplemented animals showed an increase in *Tnf* transcription. This finding is quite important since TNF plays an important role in controlling intracellular pathogens infection [55]. The presence of IFN-γ characterizes the development of a Th1 response, an effect of great importance on antiviral vaccines. IFN-γ is one of the most potent macrophage activators, and together with TNF and IL-12, it is a pro-inflammatory cytokine that promotes cell-mediated immunity [56]. The significantly higher levels of *Ifng* mRNA transcription observed in splenocytes stimulated with Zymosan and LPS from the *C. intermedia*-supplemented groups suggest that this cytokine may play a role in modulating vaccine antibody production.

We observed significantly higher levels of *Il4* mRNA transcription by splenocytes from *C. intermedia*-supplemented mice compared to *S. boulardii* and control group animals. This increase in *Il4* mRNA suggests that the transcription of this cytokine may have had a role in probiotic modulation, especially as significantly greater levels of total IgG were detected in the sera of the *C. intermedia*-supplemented animals. Besides inducing an upregulation in cytokine transcription, it was also observed an increase in the mRNA levels of transcription factors. Bcl6 is a critical transcription factor for both innate and adaptive immunity, exerting regulatory control over lymphoid and myeloid immune cells [57,58]. These processes are modulated by the repression of key genes, including *BLIMP1*, *TP53*, *ATR*, and *BCL2*, among others [59]. Moreover, Bcl6 plays an essential role in the function of follicular helper CD4+ T-cells (Tfh) and follicular regulatory T-cells (Tfr), which facilitate the activation of antigen-specific B-cells, thereby promoting the production of high-affinity antibodies and the establishment of immunological memory. This functionality underscores the indispensability of Bcl6 in orchestrating potent immune responses, particularly in the context of vaccination [57,59]. The main role of STAT3 in immune cells is to mediate anti-inflammatory effects, restricting gene transcription of pro-inflammatory cytokines [60] and repressively impacting NFκβ signaling pathways [61]. Signaling via STAT3 is activated by several cytokines and their receptors, such as IL-2, IL-6, IL-10, IL-23, and IL-27 [62]. In the gut, these complex processes of signaling promote epithelial barrier integrity by stimulating tight junction proteins and mucin production, while also enhancing secretory IgA responses and preserving immune tolerance to commensals [63,64].

The observed results are intriguing and complex, suggesting that a multifactorial analysis is necessary for a more comprehensive understanding. Despite the absence of increased *Il4* and *Ifng* mRNA expression in splenocytes from *C. intermedia*-supplemented animals following in vitro stimulation with SARS-CoV-2, elevated levels of antigen-specific IgG and sIgA antibodies were observed. This dissociation suggests the absence of a classical Th1 or Th2 polarization and may reflect an immunomodulatory effect of the yeast, potentially promoting regulatory mechanisms that limit excessive activation of pro- or anti-inflammatory pathways [65]. The in vitro conditions may also fail to fully reproduce the complex cellular interactions and co-stimulatory signals present in vivo, such as those involving dendritic cells and follicular helper T cells, which are critical for cytokine production and B cell help [66]. Additionally, *C. intermedia* may influence antibody responses through alternative mechanisms involving cytokines such as IL-6, BAFF, APRIL, or TGF-β, which support B cell maturation and class switching independently of IL-4 and IFN-γ [67,68,69]. The immune response may also have occurred through non-canonical routes not primarily dependent on IL-4 or IFN-γ [69], or may have been resolved in vivo before splenocyte collection, leaving the cells in a functionally quiescent state, as memory T cells can be actively held in quiescence by regulatory mechanisms post-response [70]. Moreover, transient cytokine expression in vivo may not be detectable under the conditions of in vitro restimulation, while antibody titers reflect cumulative immune memory established over time [71]. Our results could indicate that *C. intermedia* supplementation influences splenic lymphocyte responsiveness, potentially reflecting immunoregulatory or refractory effects, although the underlying mechanisms remain to be clarified. This is consistent with findings that gastrointestinal-adapted *Candida* strains modulate immune cell function, potentially limiting excessive inflammation but also dampening antiviral responses [72]. Further investigation into other cytokines, such as IL-10, TGF-β, or IL-17, would be valuable to clarify the immune signature associated with *C. intermedia* supplementation.

When dealing with the *Candida* genus, it is always important to highlight that safety is crucial to determining the possibility of yeast usage as a tool to promote health, since even non-*albicans* species may cause infections (including *S. cerevisiae*) [73]. Most of the infections (63–70%) related to *Candida* spp. are caused by *C. albicans*, and the rest are associated with other 18–30 species classified in *Candida* genus (non-*albicans*) that comprises around 200 species, such as *C. glabrata*, *C. tropicalis*, *C. parapsilosis*, *C. krusei*, *C. auris* [73,74]. Although *C. intermedia* has traditionally been considered a non-pathogenic species, it is important to acknowledge the possibility of opportunistic behavior under certain conditions in immunocompromised individuals [75,76]. Infections caused by this yeast are rare and poorly documented in the literature. Indeed, a search performed in the PubMed^®^ (NCBI) database using the terms “*Candida intermedia*”, “infection” and “candidemia” retrieved fewer than eight reports of candidemia cases over a period of more than 70 years (1954–2025). These isolated cases have been reported in a limited number of countries, including Brazil [77], Japan [75], Qatar [78], Taiwan [76], and Iran [79]. In Iran and Qatar, reports of *C. intermedia* candidemia involved neonates, children, and patients admitted to intensive care units; in Japan and Taiwan, cases were described in elderly individuals with comorbidities such as diabetes or in those who had undergone medical interventions including catheter implantation; while in Brazil, although isolates from infected patients have been reported, no further details on their clinical origin are available. Notably, when clinical isolates of *C. intermedia* were tested against antifungal agents such as fluconazole and amphotericin B, regardless of their geographic origin, they generally did not exhibit resistance profiles [80], suggesting that treatment with conventional antifungals remains effective. This evidence suggests that while opportunistic infections may occur, they appear to be exceedingly uncommon. Nevertheless, thorough safety evaluations are essential before considering *C. intermedia* as a candidate for probiotic applications. Indeed, even strains of *S. boulardii*, commonly regarded as safe probiotics, have been shown to exhibit opportunistic behavior in vulnerable hosts [81].

On the other hand, *C. intermedia* has already been isolated from naturally fermented foods and beverages, participating in mixed fermentation processes such as those involved in sausage, cheese, fruit-based ice-creams, and *Cauim* production [82,83,84]. Furthermore, a presumption of safety regarding the use of *C. intermedia* in fermented food products was published in the Bulletin of the International Dairy Federation 495/2018 [85], and, in our study, no deaths or apparent signs of yeast infection were observed in the supplemented animals. To the best of our knowledge, this study is the first to administer *C. intermedia* in animal models under controlled conditions; therefore, there are insufficient data in the literature to fully establish the safety of its administration. Still, it should be emphasized that the *C. intermedia* strain used here was isolated from flowers (orchids, *Aspasia lunata*), reinforcing its non-clinical origin, and no suggestive evidence of pathogenic behavior was observed under the conditions tested.

The presence of *C. intermedia* in the gastrointestinal microbiota may provide functional benefits to the host by contributing to the metabolism of dietary carbohydrates that are otherwise poorly digested by human enzymes. This yeast harbors an active *LAC* gene cluster, including *LAC4* (encoding β-galactosidase) and *LAC12* (encoding a lactose permease), enabling the uptake and hydrolysis of lactose into glucose and galactose [86]. In individuals with lactose malabsorption, this metabolic capacity may reduce the accumulation of undigested lactose in the colon, thereby alleviating gastrointestinal symptoms such as bloating and diarrhea [87,88]. Similarly, *C. intermedia* is capable of metabolizing xylose, a pentose sugar derived from dietary hemicelluloses, through the action of key genes such as *XYL1* (xylose reductase), *XYL2* (xylitol dehydrogenase), and *XYL3* (xylulokinase), which together enable its assimilation via the pentose phosphate pathway [89]. Xylose is commonly found in plant-based foods rich in hemicellulose, including whole grains, legumes, berries, and some vegetables. The microbial degradation of lactose and xylose by *C. intermedia* may result in the production of short-chain fatty acids (SCFAs), such as acetate, propionate, and butyrate, which are known to promote intestinal barrier function, expand regulatory T cell populations, and modulate inflammatory responses [90,91]. Additionally, the consumption of these substrates by commensal yeasts may reduce nutrient availability for pathogenic bacteria, contributing to colonization resistance and microbial balance [92].

Besides the influence on humoral and cellular responses, we investigated changes in the GIT microbiome caused by *C. intermedia* supplementation. Although based on pooled samples, the microbiota findings provide relevant exploratory evidence of the impact of *C. intermedia* on gut microbial composition. The intestinal microbiome, composed of several microorganisms, plays an essential role in host immunity and makes great contributions to the host’s health [1]. Differences in the Firmicutes/Bacteroidetes ratio were observed in the microbiome of *C. intermedia*-supplemented mice, with increased levels of Bacteroidetes and lowered levels of Firmicutes. The increase in Bacteroidetes abundance, especially for the *Bacteroides* genus, may be related to the utilization of mannan available on *Candida* cell walls through degrading enzymes (mannanases and mannosidases) expressed by *Bacteroides* spp., serving as a nutrient (carbon) source for the bacteria [93]. *Bacteroides* spp. LPS are described as showing impaired or even inhibitory capacity to elicit an inflammatory response, and a decrease in its abundance in the gut microbiome often results in an augmented population of pro-inflammatory bacteria [94]. They also release extracellular vesicles containing immunomodulatory molecules such as capsular polysaccharides and modified lipopolysaccharides, which interact with host immune cells and promote balanced immune activation without triggering excessive inflammation [95]. The enriched presence of certain Bacteroidetes (*Prevotella* spp. and *Bacteroides* spp.) with anti-inflammatory properties was linked to fewer adverse effects after vaccination with COVID-19 vaccines and associated with the highest antibody titers in some cases [96], correlating with our results. However, this positive association is not a consensus in the literature. Commensal bacteria, such as *B. fragilis*, can modulate dendritic cell maturation and cytokine production, enhancing antigen presentation and the priming of adaptive immune responses [97].

*Bacteroides* spp. stimulate secretory IgA production, which enhances mucosal immune surveillance and may facilitate early antigen capture and presentation [98]. Their metabolic activity contributes to the production of short-chain fatty acids and other immunologically active metabolites that help shape the mucosal immune environment. *Bacteroides* spp. also engage pattern recognition receptors such as TLR2, TLR4, and NOD2 in a controlled manner, resulting in low-level immune priming that can improve responsiveness to immunization [99], as demonstrated in He et al. [100] for COVID-19 vaccination. Furthermore, they are important producers of essential vitamins and cofactors, including B vitamins and vitamin K, which support immune cell proliferation and differentiation [101]. While positive correlations between the presence of *Bacteroides* and enhanced vaccine responses have been reported, some studies have also associated a high abundance of bacteria from this genus with reduced immunogenicity compared to individuals with different microbiota compositions [94,102]. These findings suggest that the relationship between the microbiota and vaccine efficacy remains an area requiring further investigation, as current evidence indicates that such associations may be both species-specific and vaccine-specific.

Bacterial species can take advantage of the biofilm created by *Candida* and adhere to it to thrive under conditions that would be harsh for them in GIT [93,103]. It was demonstrated for *Clostridium perfringens* and *C. difficile* that these bacteria benefit from the microenvironment and microbial interactions when co-cultured with *Candida albicans* [103], and in our study, this positive interaction between *Candida-Clostridium* may be the answer to enhanced levels of *Clostridium* spp. in the microbiome of *C. intermedia*-supplemented mice. *Clostridium* species may contribute to enhanced immune responses and influence vaccine efficacy through multiple mechanisms. These bacteria maintain intestinal homeostasis by fermenting dietary fibers into short-chain fatty acids (SCFAs), particularly butyrate, which exerts anti-inflammatory effects, promotes epithelial barrier integrity, and induces the expansion of regulatory T cells (Tregs), essential for immune tolerance and inflammation control [104,105]. Additionally, *Clostridium* species produce secondary bile acids such as deoxycholic acid (DCA), lithocholic acid (LCA), and ursodeoxycholic acid (UDCA), which modulate immune cell differentiation and inhibit the growth of pathogenic microorganisms [106,107]. These immunomodulatory properties are relevant in the context of vaccination, as microbial metabolites have been shown to modulate dendritic cell activity, promote the maturation of antigen-presenting cells, support the development of gut-resident and systemic memory T cells, and enhance antibody production, all of which contribute to durable vaccine-induced immunity [108,109]. The abundance of *Lactobacillus* spp. was lower in the microbiome of yeast-supplemented mice, which may be due to the competition between yeasts and bacteria for the same metabolic niches throughout the gastrointestinal tract [74]; however, further studies are needed to prove this linkage.

Finally, several mechanisms affect the response to vaccines, including vaccine formulation, dose, immunization route, vaccination schedule, the host immune system, and the gut microbiota [110]. The modulation of resident microbiota by probiotics is a factor that may improve vaccine immunogenicity [110,111]; thus we suggest that the alterations detected in the microbiomes of the supplemented mice could be one of the key factors associated with the increase in the humoral response detected in these groups. From a broader perspective, the effects of *C. intermedia* supplementation observed here might suggest a potential to support balanced immune responses. However, whether this could translate into protection against infections, the mitigation of excessive inflammation, or recovery in conditions such as inflammatory bowel disease, post-antibiotic dysbiosis, or viral infections remains to be determined. Although *C. intermedia* has long been recognized for its biotechnological potential in the conversion of lactose and xylose into value-added products such as ethanol and xylitol [112], only with the present study new applications have been proposed for this yeast, highlighting its possible role as an immunobiotic capable of modulating vaccine-induced immune responses.

In this study, we recognize the limitation of using pooled samples for both serological and microbiota analyses, which precludes statistical evaluation of inter-individual variability and may reduce the resolution of biological differences between animals. Future studies are therefore planned to incorporate individual-level analyses, with larger sample sizes, to validate and expand upon the trends observed here. Nevertheless, we emphasize that the pooling strategy adopted in this exploratory work provided a practical and efficient means to capture the overall group-level effects of *C. intermedia* supplementation. Importantly, the pooling approach did not compromise the central conclusions, but instead enabled a robust first demonstration of the probiotic potential of *C. intermedia* in modulating immune responses and gut microbiota composition in the context of vaccination.

In summary, we demonstrated in this study that supplementation with the yeast *C. intermedia* ORQ001 can affect the expression of genes related to immune response in macrophages, modulate the GIT bacterial microbiota, and enhance the specific humoral response after vaccination. Specifically, we observed a heightened vaccine-induced immune response, evidenced by increased levels of total IgG and sIgA, as well as elevated transcription of the cytokines *Il4*, *Tnf*, and *Ifng*, and transcription factors *Bcl6* and *Stat3* in immune cells cultured in vitro. For the first time, *C. intermedia* is proposed as a microorganism capable of modulating the immune system, showing a positive impact on the immune response of mice experimentally vaccinated with inactivated SARS-CoV-2. Our in vivo findings complement previous in vitro studies that had already suggested its probiotic activity, encouraging further research.

As a future perspective, it will be important to investigate whether the immunomodulatory effects of *C. intermedia* supplementation observed in this study extend to other vaccination models. Subsequent experiments will be directed toward evaluating immune responses in animals supplemented with *C. intermedia* and immunized with vaccines specifically designed for the protection of animal infections, as well as with different classes of vaccines, including recombinant subunit vaccines, toxoid-based formulations, and emerging platforms such as mRNA vaccines. Such studies will be essential to determine the breadth and consistency of the probiotic effect of *C. intermedia* across diverse immunization strategies.

## Figures and Tables

**Table 1:** Experimental design of the groups of animals used in this experiment.

Group	Animals (*n*)	Supplementation	Vaccine	Euthanasia (Experimental Day)
A	10	-	-	0
B	10	*C. intermedia*	-	0
C	10	*S. boulardii*	-	0
D	10	-	-	42
E	10	*C. intermedia*	-	42
F	10	*S. boulardii*	-	42
G	10	-	Inactivated SARS-CoV-2 virus	42
H	10	*C. intermedia*	Inactivated SARS-CoV-2 virus	42
I	10	*S. boulardii*	Inactivated SARS-CoV-2 virus	42

## Data Availability

The data underlying this article are available in the article and in its online supplementary material.

## Supplementary Materials

The following supporting information can be downloaded at: https://www.mdpi.com/article/10.3390/jof11090685/s1, Figure S1. Schedule of animal experimentation. The experimental plan was conducted until day 42, consisting of a collection of blood and fecal samples every 7 days, supplementation with *C. intermedia* or *S. boulardii* by oral administration (days −5 to −1, and 16 to 20), and vaccination with two doses of inactivated SARS-CoV-2 virus (days 0 and 21); Table S1. Primer sequences for qPCR.; Table S2. qPCR analysis of mRNA expression in RAW 264.7 macrophages stimulated with *C. intermedia* and its derivatives; Table S3. qPCR analysis of mRNA expression in RAW 264.7 macrophages stimulated with *S. boulardii* and its derivatives; Table S4. mRNA expression in splenocytes from mice in the non-supplemented group (Day 0), evaluated by qPCR; Table S5. mRNA expression in splenocytes from mice in the *C. intermedia*-supplemented group (Day 0), evaluated by qPCR; Table S6. mRNA expression in splenocytes from mice in the *S. boulardii*-supplemented group (Day 0), evaluated by qPCR; Table S7. mRNA expression in splenocytes from unvaccinated and vaccinated mice (Day 42) stimulated with SARS-CoV-2, evaluated by qPCR. References [113,114,115,116,117,118] are cited in the Supplementary Materials.
