# Supplementary material for: Candida intermedia Supplementation Enhances Immune Response and Modulates the Gut Microbiome in SARS-CoV-2 Vaccinated Mice
Source: J Fungi (Basel). 2025 Sep 20;11(9):685. doi: 10.3390/jof11090685 (PMC12470568; doi:10.3390/jof11090685)
Supplement: Supplementary file 1 [file jof-11-00685-s001.zip › jof-3768249-supplementary.pdf]

Supplementary Material

Candida intermedia supplementation enhances immune response and modulates the gut microbiome in SARS-CoV-2 vaccinated mice

Renan E A Piraine[1,2*], Neida L Conrad[1], Vitória S Gonçalves[1], Jeferson V Ramos[1], Júlia L Froldi[2], Fausto Almeida[2], Fábio P L Leite[1]

[1]Microbiology laboratory, Technological Development Center, Federal University of Pelotas, Pelotas, Brazil

[2]Department of Biochemistry and Immunology, Ribeirão Preto Medical School, University of São Paulo, Ribeirão Preto, São Paulo, Brazil

***Corresponding author:** Dr. Renan Eugênio Araujo Piraine, renanbiotec@gmail.com

2. Material and methods

2.3.1. Animals, supplementation, and vaccination

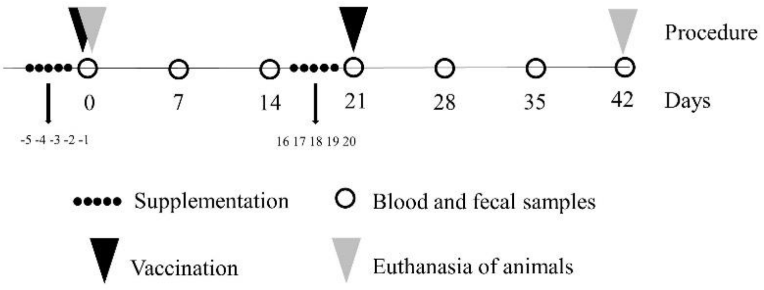

Figure S1 Schedule of animal experimentation. The experimental plan was conducted until day 42, consisting of a collection of blood and fecal samples every 7 days, supplementation with *C. intermedia* or *S. boulardii* by oral administration (days -5 to -1, and 16 to 20), and vaccination with two doses of inactivated SARS-CoV-2 virus (days 0 and 21)

2.3.3. Quantitative Real-time PCR analysis of cytokines and transcription factors genes

Table S1. Primer sequences for qPCR

Gene	Forward (5' – 3')	Reverse (5' – 3')	Reference
Actb	AGAGGGAAATCGTGCGTGAC	CAATAGTGATGACCTGGCCGT	(Cardona et al., 2003)
Il2	CCTGAGCAGGATGGAGAATTACA	TCCAGAACATGCCGCAGAG	(Cardona et al., 2003)
Il4	CTAGTTGTCATCCTGCTCTTCTTT	CTTTAGGCTTTCCAGGAAGTCTTT	(Cardona et al., 2003)
Il12p40	AGCACCAGCTTCTTCATCAGG	CCTTTCTGGTTACACCCCTCC	(Cardona et al., 2003)
Il13	TCTTGCTTGCCTTGGTGGTC	GGTCTTGTGTGATGTTGCTCAGC	(Jones et al., 2010)
Il23	CCTTCTCCGTTCCAAGATCCT	ACTAAGGGCTCAGTCAGAGTTGC T	(Lin et al., 2012)
Tnf	CATCTTCTCAAAATTCGAGTGACA A	TGGGAGTAGACAAGGTACAACCC	(Cardona et al., 2003)
Ifng	AGCGGCTGACTGAACTCAGATTG TAG	GTCACAGTTTTCAGCTGTATAGGG	(Cardona et al., 2003)
Nfκb	AGTGCAAAGGAAACGCCAGAAG	GCCAGGGCTTCCGGTACTC	(Nakata et al., 2009)
Bcl6	GCCGGCTCAATAATCTCGTGAAC AGGTCC	CCAGCAGTATGGAGGCACATCTC TGTATGC	(Karnowski et al., 2012)
Stat3	TCATGGGTTTCATCAGCAAG	GTCCTTTTCCACCCAAGTGA	(Ray et al., 2014)

3. Results

Table S2. qPCR analysis of mRNA expression in RAW 264.7 macrophages stimulated with *C. intermedia* and its derivatives

Stimulus	Target gene	Fold-change (log_2)	Biological effect
Viable cells of *C. intermedia*	*IL2*	0.68*	Upregulated
	IL4	2.94*	Upregulated
	IL13	1.23*	Upregulated
	IL23	- 0.47	-
	Tnf	- 0.49*	Downregulated
	Nfκb	0.05	-
	Bcl6	-0.01	-
	Stat3	- 1.79*	Downregulated
Heat-killed cells of *C. intermedia*	*IL2*	- 1.04*	Downregulated
	IL4	0.21	-
	IL13	- 0.50	-
	IL23	0.12	-
	Tnf	1.31*	Upregulated
	Nfκb	0.59	Upregulated
	Bcl6	2.03*	Upregulated
	Stat3	0.68*	Upregulated
Culture supernatant of *C. intermedia*	*IL2*	- 1.38*	Downregulated
	IL4	0.32	-
	IL13	- 0.96*	Downregulated
	IL23	- 0.49	-
	Tnf	0.45*	Upregulated
	Nfκb	- 0.23	-
	Bcl6	0.24	-
	Stat3	- 0.14	-
C. intermedia DNA	*IL2*	- 2.06*	Downregulated
	IL4	0.58*	Upregulated
	IL13	- 1.04*	Downregulated
	IL23	0.04	-
	Tnf	- 1.20*	Downregulated
	Nfκb	- 0.60	-
	Bcl6	- 0.25	-
	Stat3	- 0.71*	Downregulated

"*" indicates a statistically significant difference compared to basal mRNA expression.
"–" indicates no upregulation or downregulation; mRNA expression remained at basal levels.
Boxes marked in blue indicate upregulation; boxes marked in red indicate downregulation.
Fold-change (log_2) was calculated based on mean 2^ΔΔCt values obtained from qPCR experiments

Table S3. qPCR analysis of mRNA expression in RAW 264.7 macrophages stimulated with *S. boulardii* and its derivatives

Stimulus	Target gene	Fold-change ($\log_2$)	Biological effect
Viable cells of *S. boulardii*	*IL2*	3.11*	Upregulated
	IL4	1.61*	Upregulated
	IL13	3.13*	Upregulated
	IL23	2.90*	Upregulated
	Tnf	- 0.31	-
	Nfκb	- 0.23	-
	Bcl6	0.21	-
	Stat3	- 0.13	-
Heat-killed cells of *S. boulardii*	*IL2*	- 0.85*	Downregulated
	IL4	- 0.04	-
	IL13	- 0.04	-
	IL23	0.29	-
	Tnf	0.31	-
	Nfκb	0.70*	Upregulated
	Bcl6	1.73*	Upregulated
	Stat3	0.80*	Upregulated
Culture supernatant of *S. boulardii*	*IL2*	- 0.97*	Downregulated
	IL4	0.37	-
	IL13	- 0.31	-
	IL23	- 0.04	-
	Tnf	- 1.79*	Downregulated
	Nfκb	- 0.57	-
	Bcl6	0.55*	Upregulated
	Stat3	0.01	-
S. boulardii DNA	*IL2*	0.82*	Upregulated
	IL4	- 0.36	-
	IL13	- 1.54*	Downregulated
	IL23	- 1.20*	Downregulated
	Tnf	- 0.40	-
	Nfκb	- 0.40	-
	Bcl6	- 0.76*	Downregulated
	Stat3	- 0.68*	Downregulated

"*" indicates a statistically significant difference compared to basal mRNA expression.
"–" indicates no upregulation or downregulation; mRNA expression remained at basal levels.
Boxes marked in blue indicate upregulation; boxes marked in red indicate downregulation.
Fold-change ($\log_2$) was calculated based on mean 2^ΔΔCt values obtained from qPCR experiments

Table S4. mRNA expression in splenocytes from mice in the non-supplemented group (Day 0), evaluated by qPCR

Stimulus	Target gene	Fold-change (log_2)	Biological effect
Zymosan	*Il4*	- 0.70	-
	Il12	- 0.12	-
	Il23	- 1.00	-
	Tnf	2.03*	Upregulated
	Ifng	2.93*	Upregulated
	Nfκb	- 0.06	-
	Bcl6	- 0.04	-
	Stat3	0.50*	Upregulated
LPS	*Il4*	- 1.20*	Downregulated
	Il12	- 2.39*	Downregulated
	Il23	- 1.51*	Downregulated
	Tnf	0.62	-
	Ifng	0.70*	Upregulated
	Nfκb	- 0.09	-
	Bcl6	- 0.50*	Downregulated
	Stat3	- 0.23	-

"*" indicates a statistically significant difference compared to basal mRNA expression.
"–" indicates no upregulation or downregulation; mRNA expression remained at basal levels.
Boxes marked in blue indicate upregulation; boxes marked in red indicate downregulation.
Fold-change (log_2) was calculated based on mean 2^ΔΔCt values obtained from qPCR experiments

Table S5. mRNA expression in splenocytes from mice in the *C. intermedia*-supplemented group (Day 0), evaluated by qPCR

Stimulus	Target gene	Fold-change (log_2)	Biological effect
Zymosan	*Il4*	1.02*	Upregulated
	Il12	1.22*	Upregulated
	Il23	- 0.31	-
	Tnf	3.39*	Upregulated
	Ifng	5.47*	Upregulated
	Nfκb	0.08	-
	Bcl6	1.14*	Upregulated
	Stat3	2.63*	Upregulated
LPS	*Il4*	0.95*	Upregulated
	Il12	- 0.85	-
	Il23	- 0.61	-
	Tnf	1.87*	Upregulated
	Ifng	5.05*	Upregulated
	Nfκb	0.68*	Upregulated
	Bcl6	1.50*	Upregulated
	Stat3	1.99*	Upregulated

"*" indicates a statistically significant difference compared to basal mRNA expression.
"–" indicates no upregulation or downregulation; mRNA expression remained at basal levels.
Boxes marked in blue indicate upregulation; boxes marked in red indicate downregulation.
Fold-change (log_2) was calculated based on mean 2^ΔΔCt values obtained from qPCR experiments

Table S6. mRNA expression in splenocytes from mice in the *S. boulardii*-supplemented group (Day 0), evaluated by qPCR

Stimulus	Target gene	Fold-change (log_2)	Biological effect
Zymosan	*Il4*	0.59	-
	Il12	- 0.49	-
	Il23	0.82*	Upregulated
	Tnf	2.25*	Upregulated
	Ifng	3.96*	Upregulated
	Nfκb	- 0.18	-
	Bcl6	0.34	-
	Stat3	0.49	-
LPS	*Il4*	0.32	-
	Il12	- 1.57*	Downregulated
	Il23	- 0.22	-
	Tnf	0.91*	Upregulated
	Ifng	1.99*	Upregulated
	Nfκb	- 0.32	-
	Bcl6	0.76*	Upregulated
	Stat3	1.31*	Upregulated

"*" indicates a statistically significant difference compared to basal mRNA expression.
"–" indicates no upregulation or downregulation; mRNA expression remained at basal levels.
Boxes marked in blue indicate upregulation; boxes marked in red indicate downregulation.
Fold-change (log_2) was calculated based on mean 2^ΔΔCt values obtained from qPCR experiments

Table S7. mRNA expression in splenocytes from non-vaccinated and vaccinated mice (Day 42) stimulated with SARS-CoV-2, evaluated by qPCR

Experimental group	Vaccination status	Target gene	Fold-change (log_2)	Biological effect
Non-supplemented	Non-vaccinated	*II4*	- 4.84*	Downregulated
		Tnf	0.12	-
		Ifng	0.70*	Upregulated
Non-supplemented	Vaccinated	*II4*	- 0.57*	Downregulated
		Tnf	0.01	-
		Ifng	1.40*	Upregulated
C. intermedia-supplemented	Non-vaccinated	*II4*	- 0.27*	Downregulated
		Tnf	- 0.03	-
		Ifng	0.25*	Upregulated
C. intermedia-supplemented	Vaccinated	*II4*	- 0.28*	Downregulated
		Tnf	- 0.02	-
		Ifng	- 0.28	-
S. boulardii-supplemented	Non-vaccinated	*II4*	- 1.20*	Downregulated
		Tnf	0.08	-
		Ifng	- 0.63	Downregulated
S. boulardii-supplemented	Vaccinated	*II4*	- 1.45*	Downregulated
		Tnf	0.06	-
		Ifng	- 0.11	-

"*" indicates a statistically significant difference compared to basal mRNA expression.
"–" indicates no upregulation or downregulation; mRNA expression remained at basal levels.
Boxes marked in blue indicate upregulation; boxes marked in red indicate downregulation.
Fold-change (log_2) was calculated based on mean 2^ΔΔCt values obtained from qPCR experiments

4. References

Cardona, P.J.; Gordillo, S.; Díaz, J.; Tapia, G.; Amat, I.; Pallarés, Á.; Vilaplana, C.; Ariza, A.; Ausina, V. Widespread bronchogenic dissemination makes DBA/2 mice more susceptible than C57BL/6 mice to experimental aerosol infection with *Mycobacterium tuberculosis*. *Infect. Immun.* **2003**, *71*, 5845–5854.

Jones, L.A.; Roberts, F.; Nickdel, M.B.; Brombacher, F.; McKenzie, A.N.J.; Henriquez, F.L.; Alexander, J.; Roberts, C.W. IL-33 receptor (T1/ST2) signalling is necessary to prevent the development of encephalitis in mice infected with *Toxoplasma gondii*. *Eur. J. Immunol.* **2010**, *40*, 426–436.

Karnowski, A.; Chevrier, S.; Belz, G.T.; Mount, A.; Emslie, D.; D'Costa, K.; Tarlinton, D.M.; Kallies, A.; Corcoran, L.M. B and T cells collaborate in antiviral responses via IL-6, IL-21, and transcriptional activator and coactivator, Oct2 and OBF-1. *J. Exp. Med.* **2012**, *209*, 2049–2064.

Lin, J.; Zhou, Z.; Huo, R.; Xiao, L.; Ouyang, G.; Wang, L.; Sun, Y.; Shen, B.; Li, D.; Li, N. Cyr61 induces IL-6 production by fibroblast-like synoviocytes promoting Th17 differentiation in rheumatoid arthritis. *J. Immunol.* **2012**, *188*, 5776–5784.

Nakata, M.; Itou, T.; Sakai, T. Quantitative analysis of inflammatory cytokines expression in peripheral blood mononuclear cells of the ferret (*Mustela putorius furo*) using real-time PCR. *Vet. Immunol. Immunopathol.* **2009**, *130*, 88–91.

Ray, J.P.; Marshall, H.D.; Laidlaw, B.J.; Staron, M.M.; Kaech, S.M.; Craft, J. Transcription factor STAT3 and type I interferons are corepressive insulators for differentiation of follicular helper and T helper 1 cells. *Immunity* **2014**, *40*, 367–377.
